# Effect of indobufen combined with clopidogrel on efficacy and blood TGF-β and IL-6 expression after percutaneous transluminal angioplasty of arteriovenous fistula

**DOI:** 10.1097/MD.0000000000049686

**Published:** 2026-07-17

**Authors:** Shuning Feng, Liang Li, Lijie Wang, Jiaqi He, Xiaoying Wang

**Affiliations:** a Department of Nephrology, Affiliated Hospital of Hebei University of Engineering, Handan, Hebei, China; b Department of Cardiac and Great Vascular Surgery I, Handan First Hospital, Handan, Hebei, China.

**Keywords:** arteriovenous fistula, clopidogrel, indobufen, interleukin-6, percutaneous transluminal angioplasty, restenosis, transforming growth factor-beta

## Abstract

This study aimed to investigate the effect of indobufen combined with clopidogrel compared with clopidogrel alone on the efficacy and serum inflammatory factors (transforming growth factor-beta [TGF-β], interkeukin [IL]-6) in patients after percutaneous transluminal angioplasty (PTA) for arteriovenous fistula (AVF). A retrospective cohort study was conducted, enrolling 120 patients after AVF-PTA. Patients were divided into the combination therapy group (indobufen + clopidogrel) and the conventional therapy group (clopidogrel alone) by propensity score matching, with 60 patients in each group. The clinical efficacy at 7 days postoperatively, changes in AVF blood flow volume/velocity, changes in serum TGF-β/IL-6 levels, and restenosis rates at 3 and 6 months postoperatively were compared between the 2 groups. The total clinical effective rate at 7 days postoperatively was significantly higher in the combination therapy group (91.7% vs 76.7%, *P* = .024), with more significant improvements in AVF blood flow volume and velocity (both *P* < .001). The combination therapy group showed greater reductions in serum TGF-β and IL-6 levels postoperatively (both *P* < .01). The restenosis rates were significantly lower in the combination group at 3 months (6.7% vs 20.0%, *P* = .038) and 6 months (15.0% vs 36.7%, *P* = .006) postoperatively, with higher primary patency rates (*P* = .004). Indobufen combined with clopidogrel can more effectively improve early postoperative efficacy and AVF function after AVF-PTA, significantly suppress the expression of inflammatory factors, and reduce the risk of short- to medium-term restenosis. Its mechanism may be related to synergistic antiplatelet and anti-inflammatory effects.

## 1. Introduction

Arteriovenous fistula (AVF) is the preferred long-term vascular access for patients undergoing maintenance hemodialysis. However, its long-term patency faces significant challenges, as stenosis or occlusion caused by neointimal hyperplasia (NIH) is the primary cause of AVF dysfunction, severely affecting dialysis adequacy and patient prognosis.^[1,2]^ Percutaneous transluminal angioplasty (PTA) is currently the core interventional treatment for AVF stenosis and can effectively restore the vascular lumen.^[3]^ Nevertheless, the post-PTA restenosis rate remains high, with patency rates often below 50% within 6 months after the procedure, presenting an urgent issue that requires resolution.^[4]^

The occurrence of restenosis is a pathophysiological process involving multiple factors, including endothelial injury, platelet activation and aggregation, inflammatory response, and subsequent smooth muscle cell proliferation and migration.^[5,6]^ Among these, the inflammatory response is considered one of the initiating and core mechanisms driving restenosis.^[5]^ The mechanical dilation of the PTA balloon inevitably causes vascular wall injury, triggering a local inflammatory cascade and the release of various inflammatory cytokines.^[6]^ Transforming growth factor-β (TGF-β) and interleukin-6 (IL-6) play key roles in this process. TGF-β strongly promotes extracellular matrix deposition and the transformation of smooth muscle cells into a synthetic phenotype, making it a critical factor in vascular wall fibrotic thickening. IL-6, as an important pro-inflammatory factor, exacerbates the local inflammatory state and further induces the release of other inflammatory mediators, collectively promoting neointimal formation.^[7,8]^ Therefore, inhibiting the excessive inflammatory response after PTA may be an important strategy for improving the long-term efficacy of the procedure.z

Antiplatelet therapy is the cornerstone for preventing thrombosis and restenosis after AVF-PTA. Clopidogrel, as a P2Y12 receptor antagonist, is widely used clinically to inhibit platelet activation.^[7]^ However, monotherapy may have limitations in addressing the complex vascular injury response. Indobufen is a reversible cyclooxygenase inhibitor with both antiplatelet and anti-inflammatory properties.^[9]^ Studies have shown that indobufen can not only inhibit thromboxane A2 synthesis but also exert anti-inflammatory effects by influencing leukocyte activity and the release of inflammatory factors.^[10]^ Theoretically, combining clopidogrel with indobufen may more comprehensively intervene in the pathological processes of thrombosis and inflammatory stenosis after PTA through synergistic antiplatelet and additive anti-inflammatory effects. However, to date, evidence regarding the efficacy of indobufen combined with clopidogrel after AVF-PTA, its impact on key inflammatory factors such as TGF-β and IL-6, and its association with clinical outcomes remains insufficient.

Based on this, the present study aims, through a retrospective cohort study, to evaluate the clinical efficacy and safety of indobufen combined with clopidogrel compared to clopidogrel alone in patients following AVF-PTA. The focus of the research is to observe whether the combination therapy can more effectively improve early postoperative fistula function, reduce serum levels of TGF-β and IL-6, and ultimately decrease the incidence of postoperative restenosis while enhancing long-term fistula patency rates. This will provide new evidence-based medical support for optimizing post-AVF-PTA antithrombotic and anti-inflammatory management strategies.

## 2. Methods

### 2.1. Study subjects

This study was approved by the Ethics Committee of the Affiliated Hospital of Hebei University of Engineering. This study is a retrospective cohort study. Patients who underwent AVF-PTA between June 2023 and June 2025 were retrospectively enrolled. Based on whether they had previously received indobufen combined with clopidogrel or clopidogrel alone, they were divided into 2 groups. To balance the influence of confounding factors between the 2 groups, propensity score matching was employed. Ultimately, 60 patients were included in each group with balanced baseline information.

### 2.2. Inclusion and exclusion criteria

#### 2.2.1. Inclusion criteria

(1) Patients who underwent PTA for autogenous AVF stenosis or occlusion between May 2023 and May 2025;
(2) Based on postoperative treatment regimens, patients were divided into 2 groups: the combination therapy group (regular administration of indobufen combined with clopidogrel) and the conventional therapy group (receiving conventional antiplatelet regimens such as clopidogrel alone);
(3) Age ≥ 18 years;
(4) Availability of complete and regular postoperative clinical follow-up records (at least containing key data at 1, 3, and 6 months postoperatively);
(5) Availability of complete and accessible clinical baseline data.

#### 2.2.2. Exclusion criteria

(1) Patients who failed to complete the specified follow-up or had missing key follow-up data;
(2) Patients with a known allergy to the study medications such as indobufen or clopidogrel, or those with active bleeding or a history of bleeding disorders;
(3) Patients with comorbidities that could significantly affect the study outcomes or patient safety, including active malignancy, severe cardiac, hepatic, or renal insufficiency, severe infection or inflammatory states, pregnancy, or lactation;
(4) Patients who received other treatments during the same postoperative period that might interfere with the study results, such as systemic immunosuppressants, anticoagulant therapy, or participation in other clinical trials;
(5) Patients whose PTA treatment involved special types of fistulas, such as prosthetic graft fistulas or brachial artery high-flow fistulas.

### 2.3. Treatment modalities

#### 2.3.1. Criteria for procedural success

Technical success: immediate postoperative assessment via color Doppler ultrasound showed a residual stenosis rate of < 30% in the target lesion vessel.

Clinical success: all of the following conditions were met: the AVF could be successfully used for at least 1 hemodialysis session following PTA; the pump-controlled blood flow rate during dialysis was ≥ 200 mL/minute; a distinct thrill or a continuous blowing murmur could be palpated or auscultated at the fistula anastomosis and the draining venous segment postoperatively.

#### 2.3.2. Postoperative treatment regimen^[11]^

All patients received foundational treatment after PTA. Based on different antiplatelet treatment plans, they were divided into 2 groups:

Combination therapy group: oral administration of Indobufen Tablets (Manufacturer: Hangzhou Zhongmei Huadong Pharmaceutical Co., Ltd., Approval Number: Guo Yao Zhun Zi H20163311), 0.2 g per dose, 3 times daily; concurrently with oral Clopidogrel Bisulfate Tablets (Manufacturer: Nanjing Chia Tai Tianqing Pharmaceutical Co., Ltd., Approval Number: Guo Yao Zhun Zi H20203269), 75 mg per dose, once daily. The 2 drugs were used in combination, with treatment continuing for 7 days.

Conventional therapy group (control): Oral administration of Clopidogrel Bisulfate Tablets (Manufacturer and Approval Number as above), 75 mg per dose, once daily, as monotherapy, continued for 7 days. Other foundational treatments and postoperative management measures were consistent between the 2 groups of patients.

### 2.4. Data collection

The observation indicators of this study mainly include efficacy evaluation, monitoring of AVF function, changes in serum TGF-β and IL-6 levels, and follow-up data on postoperative restenosis rates. The specific contents are as follows:

#### 2.4.1. Efficacy evaluation

Efficacy evaluation was determined based on the Chinese Expert Consensus on Vascular Access for Hemodialysis and relevant clinical symptom criteria. Within 7 days postoperatively, treatment efficacy was assessed according to the patient’s clinical manifestations and the patency status of the AVF. The efficacy criteria are as follows:

Markedly effective: after treatment, the vascular murmur of the fistula remains continuously loud, the thrill is distinct, puncture pain is significantly reduced, local induration and hematoma have completely resolved, there is no subcutaneous bruising, and the elasticity of the fistula vessels has noticeably softened. During dialysis, blood flow can reach 280 ml/min or higher.

Effective: after treatment, the vascular murmur of the fistula is loud, the thrill is palpable, puncture pain is reduced, local induration and hematoma have partially resolved, there is mild subcutaneous bruising, and the elasticity of the fistula vessels has improved but remains slightly hardened. During dialysis, blood flow ranges between 250 and 280 ml/min.

Ineffective: after treatment, there is no significant improvement in vascular murmur and thrill. The thrill is weak or absent, puncture pain is significant, local induration and hematoma have not resolved, subcutaneous bruising is evident, and the fistula vessels are hard with a lack of significant elasticity. During dialysis, blood flow is < 250 ml/min.

#### 2.4.2. AVF function

Fistula function is one of the key indicators for evaluating treatment efficacy, mainly including fistula blood flow volume and blood flow velocity. All patients underwent color Doppler ultrasound examinations preoperatively and 7 days postoperatively, measured using a SonoScape X1 color Doppler ultrasound diagnostic system. To minimize operational errors, all examinations were performed by the same experienced sonographer, ensuring data consistency and reliability.

Blood flow volume (mL/minute): measures the blood flow volume of the fistula, reflecting its patency degree and dialysis effectiveness.

Blood Flow Velocity (cm/second): evaluates the blood flow velocity of the fistula, further reflecting the vascular status and postoperative recovery of the fistula.

#### 2.4.3. Measurement of serum TGF-β and IL-6

To evaluate the inflammatory response and its impact on postoperative fistula recovery, serum levels of TGF-β and IL-6 were sampled and analyzed preoperatively and at 7 days postoperatively:

Sample collection: fasting venous blood samples were collected from all patients preoperatively and at 7 days postoperatively. These blood samples were used to determine the concentrations of TGF-β and IL-6.

Detection method: serum samples were analyzed using enzyme-linked immunosorbent assay kits. The specific kit information is as follows:

TGF-β kit: Lot No. AD16732Hu, purchased from Guangdong Andy Gene Biotechnology Co., Ltd.

IL-6 kit: Lot No. AD191209, purchased from Guangdong Andy Gene Biotechnology Co., Ltd.

Detection Purpose: by measuring serum concentrations of TGF-β and IL-6, this study aimed to assess changes in postoperative inflammatory response and further investigate its relationship with fistula recovery.

#### 2.4.4. Incidence of restenosis after PTA for AVF stenosis

The recurrence of postoperative stenosis is a critical factor affecting the long-term patency of the AVF and dialysis efficacy. To assess the long-term impact of treatment, this study conducted follow-ups within 3 and 6 months postoperatively and collected the following data:

Restenosis incidence: ultrasound or angiographic examinations were used to assess whether restenosis occurred within the fistula at 3 and 6 months postoperatively, and the degree and timing of restenosis were recorded.

Follow-up process: each patient underwent at least 1 follow-up examination within 3 and 6 months postoperatively to ensure timely detection of restenosis and necessary interventions.

Evaluation criteria: restenosis was determined if symptoms such as decreased fistula blood flow or vascular stenosis occurred within 3 and 6 months postoperatively. The incidence of restenosis and its relationship with the treatment groups were recorded through follow-up and corresponding treatment plans.

### 2.5. Statistical analysis

Data analysis in this study was performed using SPSS statistical software (IBM SPSS Statistics, version 26.0; IBM Corp.). Continuous variables conforming to a normal distribution are presented as mean ± standard deviation, and intergroup comparisons were conducted using independent samples *t*-tests. For data not conforming to a normal distribution, variables are presented as median (interquartile range), and intergroup differences were assessed using the Mann–Whitney *U* test. Categorical variables are expressed as frequencies and percentages, with intergroup comparisons performed using the chi-square test (*χ*^2^). Clinical efficacy comparisons were conducted via the chi-square test and further analyzed using ordinal logistic regression to adjust for confounding factors. Analysis of postoperative restenosis rates and patency was performed using the chi-square test and Kaplan–Meier survival analysis. Subgroup analysis employed interaction tests. A *P* value of < .05 was considered statistically significant.

## 3. Results

### 3.1. Patient baseline characteristics

A total of 120 patients were included in this study. After propensity score matching, the combination therapy group and the conventional therapy group each comprised 60 patients. Baseline characteristics are shown in Table 1. There were no significant differences between the 2 groups in terms of age, gender, body mass index, hypertension, diabetes, dialysis vintage, history of previous PTA, fistula location, stenosis type, preoperative blood flow volume, or serum levels of TGF-β and IL-6 (all *P* values > .05). These results indicate that the baseline characteristics of the 2 patient groups were balanced and exhibited good comparability.

**Table 1:** Comparison of baseline characteristics between the 2 groups after propensity score matching.

Variable	Combination Therapy Group (n = 60)	Conventional Therapy Group (n = 60)	Statistic	*P* Value
Demographics				
Age (yrs, mean ± SD)	56.8 ± 9.3	57.2 ± 8.7	*t* = 0.24	.808
Male [n (%)]	34 (56.7%)	32 (53.3%)	*χ*^2^ = 0.13	.715
BMI (kg/m^2^, mean ± SD)	23.5 ± 3.2	23.1 ± 3.4	*t* = 0.65	.519
Primary Disease & Comorbidities				
Primary Hypertension (n [%])	48 (80.0%)	46 (76.7%)	*χ*^2^ = 0.20	.657
Diabetes Mellitus (n [%])	35 (58.3%)	33 (55.0%)	*χ*^2^ = 0.14	.711
Dialysis Vintage (months, median [IQR])	42 (24, 65)	38 (22, 63)	*Z* = 0.52	.603
AVF Characteristics				
History of Prior PTA (n [%])	16 (26.7%)	18 (30.0%)	*χ*^2^ = 0.16	.689
AVF Location (Forearm, n [%])	45 (75.0%)	47 (78.3%)	*χ*^2^ = 0.19	.666
AVF Age before PTA (months, median [IQR])	28 (16, 44)	26 (14, 42)	*Z* = 0.38	.704
Stenosis Type (Anastomotic, n [%])	22 (36.7%)	20 (33.3%)	*χ*^2^ = 0.15	.701
Preoperative Stenosis Degree (%, mean ± SD)	72.5 ± 10.8	73.8 ± 11.2	*t* = 0.63	0.528
Preoperative AVF Blood Flow (mL/min, mean ± SD)	185.6 ± 42.3	180.9 ± 45.1	*t* = 0.58	.562
Laboratory Parameters				
Preoperative Serum TGF-β (pg/mL, median [IQR])	85.2 (62.1, 114.5)	88.6 (65.3, 118.0)	*Z* = 0.47	.638
Preoperative Serum IL-6 (pg/mL, median [IQR])	12.3 (8.5, 18.1)	13.0 (9.0, 19.4)	*Z* = 0.71	.478
Preoperative Hemoglobin (g/L, mean ± SD)	108.5 ± 13.2	106.8 ± 14.5	*t* = 0.66	.512
Preoperative Platelet Count (× 10^9^/L, mean ± SD)	185 ± 52	178 ± 49	*t* = 0.74	.463

AVF = arteriovenous fistula, BMI = body massi index, IQR = inerquartile range, n = number of participants, PTA = percutaneous transluminal angioplasty, SD = standard deviation.

### 3.2. Comparison of clinical efficacy

The results of the clinical efficacy evaluation at 7 days postoperatively are presented in Table 2. The overall effective rate (markedly effective + effective) in the combination therapy group was 91.7% (55/60), which was significantly higher than the 76.7% (46/60) in the conventional therapy group (*P* = .024). Specifically, in the combination therapy group, 35 patients (58.3%) showed markedly effective results and 20 patients (33.3%) were classified as effective, whereas the corresponding numbers in the conventional therapy group were 18 (30.0%) and 28 (46.7%), respectively. Furthermore, ordinal logistic regression analysis demonstrated that, after adjusting for potential confounding factors, the combination therapy regimen was an independent favorable factor associated with a higher efficacy grade (adjusted odds ratio = 2.85, 95% confidence interval: 1.42–5.72, *P* = .003).

**Table 2:** Comparison of clinical efficacy 7 days post-PTA between the 2 groups.

Efficacy Grade	Combination Therapy Group (n = 60) (n [%])	Conventional Therapy Group (n = 60) (n [%])	Statistic	*P* Value
Markedly Effective	35 (58.3%)	18 (30.0%)		
Effective	20 (33.3%)	28 (46.7%)		
Ineffective	5 (8.3%)	14 (23.3%)		
Overall Response Rate (Markedly Effective + Effective)	55 (91.7%)	46 (76.7%)	*χ*^2^ = 5.07	.024
Ordinal Logistic Regression Analysis	OR = 2.85, 95% CI: 1.42–5.72		*Z* = 3.02	.003

CI = confidence interval, n = number of participants, OR = odds ratio.

### 3.3. Changes in AVF function indicators

At 7 days postoperatively, both treatment groups showed significant improvements in AVF blood flow volume and blood flow velocity compared to preoperative levels (intragroup comparison *P* < .001). As shown in Table 3, the blood flow volume in the combination therapy group was 292.4 ± 35.1 mL/minute, which was significantly higher than the 252.7 ± 40.8 mL/minute in the conventional therapy group (*P* < .001). Concurrently, the blood flow velocity in the combination therapy group was 198.5 ± 25.4 cm/second, also significantly higher than the 176.8 ± 32.6 cm/second in the conventional therapy group (*P* < .001).

**Table 3:** Comparison of AVF function indicators before and after treatment between the 2 groups.

Indicator	Time Point	Combination Therapy Group (n = 60)	Conventional Therapy Group (n = 60)	Intergroup *P* value	Intergroup Statistic
Blood Flow (mL/min)	Preoperative	185.6 ± 42.3	180.9 ± 45.1	.562	0.58
	Postoperative Day 7	292.4 ± 35.1	252.7 ± 40.8	< .001	5.67
	Intragroup *P* Value	< .001	< .001		
	Intragroup Statistic	18.42	14.95		
Blood Flow Velocity (cm/s)	Preoperative	125.3 ± 28.7	122.1 ± 30.5	.548	0.60
	Postoperative Day 7	198.5 ± 25.4	176.8 ± 32.6	< .001	4.02
	Intragroup *P* Value	< .001	< .001		
	Intragroup Statistic	20.15	16.32		

AVF = arteriovenous fistula, n = number of participants.

### 3.4. Changes in serum inflammatory factor levels

At 7 days postoperatively, inflammatory markers decreased in both patient groups, indicating a reduction in the inflammatory response following treatment. As shown in Table 4, serum levels of TGF-β and IL-6 were significantly lower than preoperative levels in both the combination therapy and conventional therapy groups (intragroup comparison *P* < .01). Specifically, the reduction in TGF-β in the combination therapy group was –30.5 (–45.1, –18.8) pg/mL, which was significantly greater than the reduction of –13.2 (–25.4, –5.1) pg/mL in the conventional therapy group (*P* < .001). The reduction in IL-6 was also more pronounced in the combination therapy group (–5.8 pg/mL vs –3.1 pg/mL, *P* = .003).

**Table 4:** Analysis of serum TGF-β and IL-6 levels before and after treatment.

Parameter	Time Point	Combination Therapy Group (n = 60)	Conventional Therapy Group (n = 60)	Intergroup *P* Value	Intergroup Statistic
TGF-β (pg/mL)	Preoperative	85.2 (62.1, 114.5)	88.6 (65.3, 118.0)	.638	0.47
	Postoperative Day 7	52.4 (38.7, 70.2)	73.1 (55.4, 95.8)	< .001	4.82
	Δ (Post−Pre)	−30.5 (−45.1, −18.8)	−13.2 (−25.4, −5.1)	< .001	5.14
	Intragroup *P* Value	< .001	< .001		
	Intragroup Statistic	5.89	4.23		
IL-6 (pg/mL)	Preoperative	12.3 (8.5, 18.1)	13.0 (9.0, 19.4)	.478	*Z* = 0.71
	Postoperative Day 7	6.5 (4.2, 9.8)	9.7 (6.3, 14.5)	< .001	*Z* = 4.05
	Δ (Post−Pre)	−5.8 (−9.2, −3.1)	−3.1 (−6.5, −1.0)	.003	*Z* = 2.96
	Intragroup *P* Value	< .001	.001		
	Intragroup Statistic	5.41	3.46		

IL = interleukin, n = number of participants, TGF- β = transforming growth factor-beta.

### 3.5. Analysis of postoperative restenosis rate and patency

At 3 months postoperatively, the restenosis rate in the combination therapy group was 6.7% (4/60), significantly lower than the 20.0% (12/60) in the conventional therapy group (*P* = .038), as shown in Table 5. The primary patency rates were 93.3% and 80.0%, respectively. At 6 months postoperatively, the restenosis rate in the combination therapy group was 15.0% (9/60), compared to 36.7% (22/60) in the conventional therapy group (*P* = .006). Kaplan–Meier analysis revealed that the primary patency survival rate was significantly higher in the combination therapy group than in the conventional therapy group (*P* = .004), with a restenosis risk 0.41 times that of the conventional therapy group (hazard ratio = 0.41, 95% confidence interval: 0.22–0.77).

**Table 5:** Post-PTA stenosis recurrence rates and patency analysis.

Outcome Measure	Combination Therapy Group (n = 60)	Conventional Therapy Group (n = 60)	*P* Value	Statistic
Follow-up at 3 Months				
Patients with stenosis recurrence (n [%])	4 (6.7%)	12 (20.0%)	.038	*χ*^2^ = 4.32
Primary patency rate	93.30%	80.00%		
Follow-up at 6 Months				
Patients with stenosis recurrence (n [%])	9 (15.0%)	22 (36.7%)	.006	*χ*^2^ = 7.56
Patients lost to follow-up (n)	2	3		
Primary patency rate	81.70%	56.70%		
Kaplan–Meier Survival Analysis (Log-rank test)				
Median primary patency time (d)	Not reached	154	.004	*χ*^2^ = 8.25
HR for stenosis recurrence (95% CI)	0.41 (0.22–0.77)	Reference (1.00)		

CI = confidence interval, HR = hazard ratio, n = number of participants.

### 3.6. Subgroup analysis and safety

Overall, the improvement effect of combination therapy remained consistent across different populations. As shown in Table 6, combination therapy demonstrated significantly greater improvement in blood flow volume across all subgroups (*P* < .001). Regardless of whether patients had diabetes or a history of previous PTA, the magnitude of improvement in the combination therapy group was significantly superior to that in the conventional therapy group. For example, the improvement values were 98.2 ± 30.1 and 117.3 ± 33.5 mL/minute in diabetic and nondiabetic patients, respectively; in patients with a history of previous PTA, the improvement was 95.5 ± 28.7 mL/minute, all significantly better than those in the control group (*P* < .001). Interaction analysis revealed that the efficacy of combination therapy was more pronounced in patients with a history of previous PTA (*P* = .042). Regarding safety outcomes, no major bleeding events, gastrointestinal hemorrhage, intracranial hemorrhage, or bleeding-related hospitalizations were recorded in either group during the treatment and follow-up periods. No patient discontinued treatment because of bleeding complications.

**Table 6:** Subgroup efficacy analysis and safety assessment.

Subgroup	Combination Therapy Group (n = 60)	Conventional Therapy Group (n = 60)	*P* Value for Difference	*P* Value for Interaction
Overall Population	106.8 ± 32.5	71.8 ± 28.3	< .001	-
By Diabetes Status				.135
With Diabetes (n = 68)	98.2 ± 30.1	65.4 ± 25.8	< .001	
Without Diabetes (n = 52)	117.3 ± 33.5	80.1 ± 30.2	< .001	
By History of Prior PTA				.042
With Prior PTA (n = 48)	95.5 ± 28.7	58.9 ± 24.3	< .001	
Without Prior PTA (n = 72)	114.1 ± 33.9	79.5 ± 29.1	< .001	
By Stenosis Location				.318
Anastomotic (n = 42)	110.5 ± 31.8	73.6 ± 26.9	< .001	
Non-Anastomotic (n = 78)	104.9 ± 32.9	70.9 ± 29.0	< .001	

n = number of participants, PTA = percutaneous transluminal angioplasty.

## 4. Discussion

This study aimed to evaluate the efficacy of indobufen combined with clopidogrel in patients following PTA of AVF and its impact on the inflammatory factors TGF-β and IL-6. The findings indicate that combination therapy demonstrated superior effects across several key clinical outcomes compared to clopidogrel alone, particularly in improving postoperative fistula function, suppressing the inflammatory response, and reducing restenosis. These results suggest that combination therapy may optimize post-AVF-PTA management through synergistic antiplatelet and anti-inflammatory mechanisms, potentially offering a more effective treatment strategy for such patients.

Specifically, the combination therapy group showed a significantly higher overall clinical effective rate at 7 days postoperatively, along with greater improvements in AVF blood flow volume and velocity. These outcomes likely reflect the enhanced efficacy of combination therapy in improving fistula patency and restoring dialysis blood flow.^[12]^ AVF blood flow volume and velocity are critical indicators for assessing fistula function, directly impacting dialysis adequacy.^[13]^ The significant improvements observed in the combination therapy group may be attributed to the combined anti-inflammatory effect of indobufen and the antiplatelet effect of clopidogrel, which may reduce postoperative inflammatory response and thrombus formation, thereby facilitating faster fistula recovery.^[14]^

Furthermore, the significant reduction in serum inflammatory factors TGF-β and IL-6 further supports the advantage of combination therapy in suppressing the postoperative inflammatory response. The inflammatory response is a key mechanism contributing to restenosis after AVF-PTA, particularly the role of TGF-β in NIH and vascular wall fibrosis, which may accelerate fistula stenosis development.^[15]^ IL-6, as a crucial pro-inflammatory factor, also plays a pivotal role in local inflammatory responses.^[16]^ The notably greater reductions in TGF-β and IL-6 observed in the combination therapy group compared to the conventional therapy group may represent one of the mechanisms underlying its improved postoperative recovery and reduced restenosis risk. Importantly, the observed reduction in TGF-β and IL-6 may not merely represent a transient anti-inflammatory effect. Previous studies have demonstrated that vascular injury-induced inflammatory responses are closely linked to subsequent NIH and vascular remodeling. TGF-β promotes extracellular matrix accumulation and vascular fibrosis, whereas IL-6 contributes to sustained inflammatory activation and smooth muscle cell proliferation. Therefore, early suppression of these inflammatory mediators following PTA may interrupt the cascade leading to pathological vascular remodeling, thereby contributing to improved long-term patency and lower restenosis rates. This biological rationale may explain the association observed in our study between early reductions in inflammatory markers and favorable vascular outcomes during follow-up.

Analysis of restenosis rates revealed that the incidence of restenosis at both 3 and 6 months postoperatively was significantly lower in the combination therapy group compared to the conventional therapy group, and primary patency rates were significantly higher. This suggests that combination therapy not only improves fistula function in the short term but may also maintain fistula patency over a longer duration by inhibiting inflammatory responses and thrombus formation.^[17]^ Kaplan–Meier survival analysis further demonstrated that combination therapy effectively prolongs the primary patency period of the fistula and reduces the risk of restenosis. This outcome may reflect the role of combination therapy in promoting vascular wall repair and suppressing inflammatory responses, thereby decreasing the occurrence of restenosis.^[18,19]^ Safety is an important consideration when intensifying antiplatelet therapy. In the present study, no major bleeding events or bleeding-related treatment discontinuations were observed during the study period. Although the sample size was relatively limited and minor bleeding events may have been underreported because of the retrospective design, these findings suggest that short-term administration of indobufen combined with clopidogrel demonstrated an acceptable safety profile in this cohort.

Subgroup analysis further confirmed the consistent efficacy of combination therapy across different patient populations, whether in patients with diabetes, those with a history of previous PTA, or patients with different stenosis locations. Combination therapy consistently demonstrated superior efficacy. Particularly for patients with a history of previous PTA, the efficacy of combination therapy was more pronounced, which may be related to its better intervention in long-term inflammatory responses and vascular repair processes.^[20]^

In addition, the generalizability of the present findings should be interpreted with caution. Post-PTA antithrombotic management strategies vary considerably across institutions, countries, and guideline frameworks. The combination regimen evaluated in this study reflects the practice pattern of our center and may not fully represent treatment approaches adopted elsewhere. Differences in patient characteristics, dialysis access surveillance protocols, procedural techniques, and postoperative medication strategies may influence treatment outcomes. Therefore, multicenter prospective studies are needed to validate whether the benefits observed in our cohort can be consistently reproduced across different healthcare settings.

While the findings of this study suggest that combination therapy demonstrates clear advantages in improving early postoperative fistula function and suppressing inflammatory responses, several limitations should be acknowledged. Firstly, as a retrospective cohort study, the research design may be susceptible to inherent selection bias and information bias. Secondly, although propensity score matching was employed to balance baseline characteristics between the 2 groups, it cannot entirely eliminate the influence of other potential confounding factors. Furthermore, while the follow-up period of 6 months provides valuable data, longer-term follow-up is necessary to further assess the sustained efficacy and safety of the combination therapy. Furthermore, the primary short-term outcome of “clinical efficacy” was based partly on clinical manifestations and fistula physical examination findings, which may introduce a degree of subjectivity despite the use of predefined evaluation criteria. Consequently, the results of this endpoint should be interpreted alongside more objective indicators. In the present study, Doppler ultrasound-derived blood flow parameters, restenosis rates, and primary patency outcomes provide stronger objective evidence supporting the therapeutic benefit of indobufen combined with clopidogrel.

In summary, the results of this study indicate that indobufen combined with clopidogrel can effectively improve fistula function, significantly suppress the expression of inflammatory factors, and reduce the incidence of postoperative restenosis in patients after AVF-PTA. This treatment regimen, through its dual action of antiplatelet and anti-inflammatory effects, may offer a more comprehensive therapeutic strategy. Future prospective, randomized controlled trials are warranted to further validate these findings and provide additional evidence for optimizing post-AVF-PTA management.

## Author contributions

**Conceptualization:** Shuning Feng, Liang Li, Lijie Wang, Jiaqi He, Xiaoying Wang.

**Data curation:** Shuning Feng, Liang Li, Lijie Wang, Jiaqi He, Xiaoying Wang.

**Formal analysis:** Shuning Feng, Liang Li, Lijie Wang, Jiaqi He, Xiaoying Wang.

**Funding acquisition:** Shuning Feng, Xiaoying Wang.

**Investigation:** Xiaoying Wang.

**Writing** – **original draft:** Xiaoying Wang.

**Writing** – **review & editing:** Xiaoying Wang.
